# An Act to Amend and Re-Enact Sections 1767 and 1774 of the Code of Virginia

**Published:** 1894-06

**Authors:** J. Bell Bigger

**Affiliations:** Clerk of House of Delegates, and Keeper of the Rolls of Virginia.


ARTICLE III.

### AN ACT TO AMEND AND RE-ENACT SECTIONS 1767 AND 1774 OF THE CODE OF VIRGINIA.

---

As amended and re-enacted by an act entitled "An act approved January twenty-eighth, eighteen hundred and ninety, entitled 'An act to amend and re-enact Sections seventeen hundred and sixty-seven and seventeen hundred and seventy-four of Chapter seventy-nine, Code of Virginia, relating to the practice of dentistry,'" approved March 2, 1894.

*Be it Enacted by the General Assembly of Virginia:*

That Sections one thousand seven hundred and sixty-nine and one thousand seven hundred and seventy-two of the Code of Virginia, and also Sections one thousand seven hundred and sixty-seven and one thousand seven hundred and seventy-four of said Code, as amended and re-enacted by an act entitled "An act to amend and re-enact Sections one thousand seven hundred and sixty-seven and one thousand seven hundred and seventy-four of Chapter seventy-nine, Code or virginia, relating to the practice of dentistry," be amended and re-enacted so as to read as follows :

SECTION 1767. WHO MAY PRACTICE DENTISTRY.—From and after the passage of this act, it shall be unlawful for any person to engage in the practice of dentistry in the Commonwealth of Virginia, or to assist in the practice of dentistry as either an assistant or employee, or to receive license from any commissioner of revenue, unless such person has graduated and received a diploma from the faculty of a reputable institution where this specialty is taught, and chartered under the authority of some one of the United States, recognized as of good repute by the Board of Examiners, duly appointed under the provisions of section one thousand seven hundred and sixty-eight of this chapter, and shall have obtained a certificate from said Board of Examiners as provided in section one thousand seven hundred and sixty-nine of said chapter; provided, that persons who held license to practice dentistry in this Commonwealth on the twenty-eighth day of January, eighteen hundred and ninety, and have complied with the requirements of section one thousand seven hundred and seventy-four, shall be otherwise exempt from the provisions of this section; and provided further, that nothing contained in this section shall prevent any authorized physician or surgeon, or other person; from extracting teeth for any one suffering from toothache.

SECTION 1769. THEIR DUTIES.—It shall be the duty of this Board—

*First.* MEETINGS.—To meet annually at the time and place of meeting of the Virginia State Dental Association, or at such other time or place as the board shall agree upon, to conduct the examination of applicants. They shall also meet for the same purpose at the call of any four members of the Board, at such time and place as may be designated by said members. Thirty days' notice of the meetings shall be given by advertising in at least two of the daily papers published in the State.

*Second.* EXAMINATION OF APPLICANTS, ETC.—To grant a certificate of ability to practice dentistry to all applicants who undergo a satisfactory examination, and receive at least four affirmative votes, which certificates shall be signed by the members of the Board; and be stamped with a suitable seal (which they may adopt).

*Third.* REGISTRY.—To keep a book in which shall be registered the name and quslification (as far as practicable). of every person to whom such a certificate is granted.

*Fourth.* TEMPORARY CERTIFCATES.—Any member of the Board, designated by the President thereof, may, upon presentation by any applicant of the evidence of the necessary qualifications to practice dentistry under this chapter, grant a temporary license to practice until the next meeting of the board, and no longer; provided, that no such temporary license shall be granted to any person who has been rejected on an examination by the Board. All such temporary licenses shall be signed by the Secretary of the Board.

SECTION 1772. PENALTIES.—Any person who shall in violation of this chapter practice dentistry in this State, shall, on conviction thereof, be fined not less than fifty nor more than two hundred dollars, and shall not be entitled to any fee for services rendered, and if a fee shall have been paid, the patient may recover back the same. On the trial of any person charged with the violation of any of the provisions of this chapter, it shall be incumbent on the defendant to show that he has authority under the law to practice dentistry in this State in order to relieve himself from the penalties herein prescribed.

Any commissioner of the revenue who shall in violation of section seventeen hundred and sixty-seven issue a license to any person not authorized to practice dentistry by this chapter, shall, upon conviction thereof, be fined not less than twenty, nor more than fifty dollars, and no license issued by any commissioner in violation of this chapter shall be valid.

SECTION 1774. DENTISTS REQUIRED TO REGISTER.—Every person practicing dentistry in the Commonwealth of Virginia at the time of the passage of this act, shall register his name and postoffice address, together with the name of the college from which he is a graduate, or the length of time he has been practicing in this Commonwealth, with the Board of Examiners before renewing his license, and it shall be the duty of the Board to issue to each person so registered, a certificate of registration, stamped with the seal of the Board, but no fee shall be collected from persons so registering.

Every person holding a certificate of qualification or registration from the Board of examiners at the time of the passage of this act, shall, within sixty days therefrom, have his certificate recorded in the clerk's office of the county or corporation court of every county or city in which he proposes to practice, and if in the city of Richmond, in the office of the clerk of the Chancery Court of said city, and every person who shall, after the passage of said act, obtain such certificate from the Board, shall have said certificate recorded in the same manner within sixty days-after receiving the same, and before receiving a license from any commissioner of the revenue.

The certificate shall be recorded in a book kept for the purpose and properly indexed.

The clerk's fee for recording such a certificate shall be fifty cents.

This act shall be in force from its passage.

A copy. J. BELL BIGGER,

*Clerk of House of Delegates, and Keeper of the Rolls of Virginia.*

March 7. 1894.

